# Experimental Tests of the Virtual Circular Genome Model for Nonenzymatic RNA Replication

**DOI:** 10.1021/jacs.3c00612

**Published:** 2023-03-24

**Authors:** Dian Ding, Lijun Zhou, Shriyaa Mittal, Jack W. Szostak

**Affiliations:** † Department of Chemistry and Chemical Biology, Harvard University, 12 Oxford Street, Cambridge, Massachusetts 02138, United States; ‡ Department of Molecular Biology and Center for Computational and Integrative Biology, Massachusetts General Hospital, 185 Cambridge Street, Boston, Massachusetts 02114, United States; § Department of Genetics, Harvard Medical School, 77 Avenue Louis Pasteur, Boston, Massachusetts 02115, United States; ∥ Department of Biochemistry and Biophysics, Perelman School of Medicine, University of Pennsylvania, Philadelphia, Pennsylvania 19104, United States; ⊥ Howard Hughes Medical Institute, Department of Chemistry, The University of Chicago, Chicago, Illinois 60637, United States

## Abstract

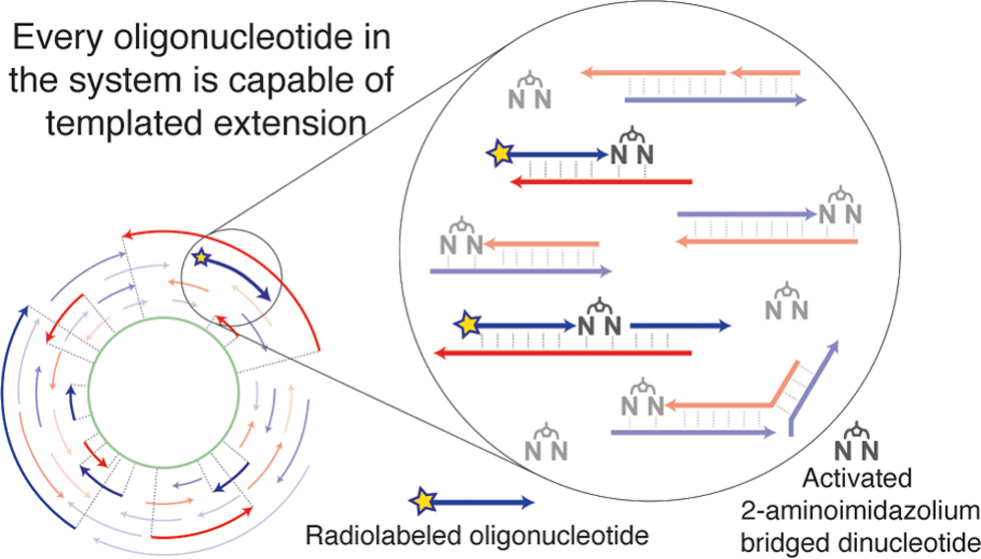

The virtual circular genome (VCG) model was proposed as a means of going beyond template copying to indefinite cycles of nonenzymatic RNA replication during the origin of life. In the VCG model, the protocellular genome is a collection of short oligonucleotides that map to both strands of a virtual circular sequence. Replication is driven by templated nonenzymatic primer extensions on a subset of kinetically trapped partially base-paired configurations, followed by the shuffling of these configurations to enable continued oligonucleotide elongation. Here, we describe initial experimental studies of the feasibility of the VCG model for replication. We designed a small 12-nucleotide model VCG and synthesized all 247 oligonucleotides of lengths 2 to 12 corresponding to this genome. We experimentally monitored the fate of individual labeled primers in the pool of VCG oligonucleotides following the addition of activated nucleotides and investigated the effect of factors such as oligonucleotide length, concentration, composition, and temperature on the extent of primer extension. We observe a surprisingly prolonged equilibration process in the VCG system that enables a considerable extent of reaction. We find that environmental fluctuations would be essential for continuous templated extension of the entire VCG system since the shortest oligonucleotides can only bind to templates at low temperatures, while the longest oligonucleotides require high-temperature spikes to escape from inactive configurations. Finally, we demonstrate that primer extension is significantly enhanced when the mix of VCG oligonucleotides is preactivated. We discuss the necessity of ongoing in situ activation chemistry for continuous and accurate VCG replication.

## Introduction

Nonenzymatic RNA replication is thought to have been an essential early step that allowed the first RNA world protocells to begin the process of Darwinian evolution. As an intermediate stage between untemplated nucleotide polymerization and ribozyme-catalyzed RNA replication, template-directed nonenzymatic replication could have enabled the replication of protocells seeded with initially random sequences. Such a chemically driven exploration of sequence space would have set the stage for the evolution of the first functional ribozymes. Although recent advances have suggested potential routes for extensive template copying by RNA primer extension, going beyond template copying to cycles of replication remains a significant challenge.^1^

The nonenzymatic copying of a long RNA strand would result in a stable duplex that must be dissociated to allow for the next round of replication. A variety of potential solutions to the strand separation problem have been suggested. Thermal denaturation can readily dissociate short RNA duplexes, but this becomes increasingly challenging with strands long enough to fold into functional ribozymes.^1^ Other environmental influences such as pH fluctuations,^2^ solvent viscosity cycles,^3^ microscale water evaporation/condensation cycles,^4−6^ or other special geological properties can potentially couple with thermocycling to facilitate strand separation. For example, heat flux across a cylindrical pore can facilitate the periodic shuffling of a complex mixture of oligonucleotides to enable ribozyme-catalyzed RNA replication through ligation.^7,8^ However, in the absence of ribozymes, the rate of template copying at reasonable concentrations is much slower than the reannealing of the separated strands, which would block primer extension. Small fractions of backbone 2′-5′ linkages or DNA were shown to lower the melting temperature,^9−11^ but they also increase the hydrolytic lability of the duplex and slow down primer extension.^12,13^ As an alternative strategy, our lab has previously demonstrated that RNA oligonucleotides can lead to toehold-mediated branch migration that can open up a segment of the duplex, allowing for strand displacement synthesis by nonenzymatic primer extension.^14^ This approach is closer to the helicase-catalyzed strand displacement that occurs at replication forks in modern biology, but other problems with nonenzymatic RNA replication remain.

The difficulties in replicating ribozyme-length sequences recently led us to consider the assembly of functional ribozymes by the ligation of shorter oligonucleotides that would be easier to replicate. Our lab has recently demonstrated that splinted ligation and loop-closing ligation can form functional ribozymes from short oligonucleotides.^15,16^ However, even the replication of shorter oligonucleotides faces problems that can lead to information loss at both ends of the sequence. First, the nonenzymatic copying of the last base of a sequence by primer extension is known to be very slow relative to the copying of internal nucleotides,^17^ which could lead to progressive loss of 3′-sequences over cycles of replication. This notorious “last base addition problem” is now understood as being due to the primary mechanism of nonenzymatic primer extension, which requires the binding of an imidazolium-bridged dinucleotide intermediate (N*N) to the template by two base pairs.^18^ With only one base pair possible at the last base of the template, binding of the bridged dinucleotide is greatly weakened, thus reducing the rate of primer extension. While an imidazole-activated mononucleotide can still perform nonenzymatic primer extension, the reaction is much slower and more error-prone.^19,20^

Maintenance of the genetic information at the 5′-end of a sequence is even more problematic since this would require a continuous supply of a specific primer, which is clearly not prebiotically plausible. As a result, information will be lost when nonenzymatic primer extension is initiated at an internal position on a template. Although ligation events could potentially salvage some internally initiated strands, this process is slow and inefficient and would be completely prevented if the 5′-end is unphosphorylated or is blocked by a nucleotide 5′-5′-pyrophosphate cap.

These problems have led others to propose that primordial genome replication occurred by a rolling circle process, in which primer extension continues many times around a circular template, spinning off a long multimeric single-stranded product.^21,22^ As in modern viroid replication, this linear product would have to be cleaved into unit-length strands, which would then have to become circularized to generate a circular template. The process would then have to repeat for the other strand. Since this process would require very extensive primer extensions in the face of the topological difficulties of replicating a small circular RNA, as well as requiring multiple ribozyme activities for cleavage and circularization, we do not consider rolling circle replication to be a viable model for nonenzymatic RNA replication.

**Figure 1:**
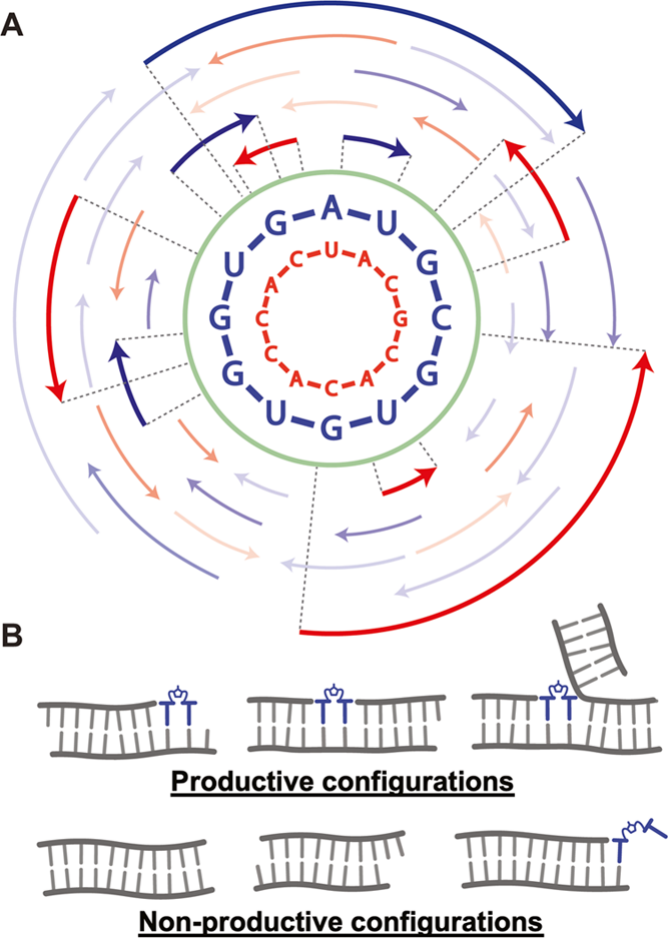
(A) Schematic illustration of the virtual circular genome model. The green circle represents the virtual genome that does not correspond to any actual oligonucleotide. A subset of the real oligonucleotides in the VCG system is illustrated as the blue and red arrows. Dotted lines, along with the bold arrows, showed how the oligomers map onto the virtual circular genome. The two complementary sequences selected for this study are shown inside the green circle. The direction from 5′ to 3′ is clockwise for the blue sequence and counterclockwise for the red sequence, which is the same direction as the arrows. Adapted from Figure 3 of ref (23) with permission under a Creative Commons Attribution 4.0 International License. Copyright 2021 Zhou et al.; Cold Spring Harbor Laboratory Press for the RNA Society. (B) Examples of productive and nonproductive configurations of annealed oligonucleotides.

The above problems led us to propose the virtual circular genome (VCG) model for prebiotically plausible nonenzymatic RNA replication.^23^ Under prebiotically plausible conditions, spontaneous untemplated^24−27^ and templated polymerization^28^ may give rise to a large diversity of short oligonucleotides, small subsets of which could then become encapsulated within lipid vesicles. As a result, each primordial protocell genome would initially consist of a unique collection of short oligonucleotides. In a fraction of such cases, oligonucleotide overlaps would occur, such that the encapsulated oligonucleotides would map onto one or both strands of one or more virtual circular sequences (Figure 1A). Since a circular genome does not have a defined start or end, copying can be initiated and terminated at any position. This genome is not represented by any actual circular molecules but is instead represented by all possible fragments from both strands of the virtual sequence. In theory, every oligonucleotide in this system can act as a primer, template, or as a downstream helper due to stacking interactions or by forming an imidazolium-bridged intermediate. Denaturation and reannealing induced by environmental fluctuations can generate kinetically trapped partially base-paired configurations,^29^ of which a productive fraction will enable primer extensions and ligations to occur (Figure 1B). Shuffling of these base-paired configurations would allow for additional elongation to occur, and RNA-mediated branch migration could also open up base-paired regions, allowing for primer extension by strand displacement synthesis. In this model, the process of genetic replication is distributed across all of the oligonucleotides of the entire system through cycles of rearrangements of base-paired configurations.

We envision the VCG system as, in effect, an assembly line where newly generated or introduced short oligonucleotides gradually become elongated to strands of roughly 10–20 nucleotides in length. Oligonucleotides of this length can then be assembled into functional ribozymes, either by splinted ligation^15^ or by iterated loop-closing ligation.^16^ These ribozyme building blocks could be the end products of one or potentially multiple virtual circular genomes replicating together in a protocell in a prebiotically plausible environment.

Here, we explore a model VCG system with a 12-nt long virtual genome represented by 247 different oligonucleotides, which range from 2 to 12 nucleotides in length. Several dimers and trimers occur multiple times in the sequence. Using radiolabeling, we monitored the fate of individual oligonucleotides in the system following the addition of activated nucleotides or bridged dinucleotides. We investigated the effect of factors including oligonucleotide length, concentration, and temperature on the primer extension yield. In the course of these studies, we discovered a surprisingly prolonged equilibration process of the oligonucleotide mix in the VCG system that enables a considerable extent of reaction. Furthermore, we found that environmental fluctuations would be essential for continuous and templated extension of the entire VCG system across different oligo lengths. Finally, we discuss the necessity of either a flow system or ongoing in situ activation chemistry for continuous and accurate VCG replication.

## Results

### Primer Extension in the VCG Mix vs on a Single Template

To begin to test the virtual circular genome model, we first selected a 12-nt virtual circular genome sequence with no secondary structure or kinetically severe stalling points such as UU sequences that are difficult to copy (Figure 1A). The sequence that we selected is represented by 247 different oligonucleotides, ranging from 2 to 12 nucleotides in length, that map to either strand of the virtual circular sequence (Table S1). Every oligonucleotide in the system can, in principle, bind to many complementary oligonucleotides, but the most thermodynamically favored pairing will be the formation of a fully base-paired duplex. To form kinetically trapped partially base-paired configurations for template copying, we used a brief (10 s) initial 90 °C pulse to disrupt all base-pairing. We expected subsequent fast cooling to trap a fraction of the oligonucleotides in metastable configurations that would allow complementary imidazolium-bridged dinucleotides to bind to a template strand next to a primer and react by primer extension (Figure 1B). Imidazolium-bridged dinucleotides can extend a primer by one nucleotide, with an activated mononucleotide displaced as the leaving group. All ten possible intermediates were supplied at the same concentration (∼1.7 mM each) for all primer extension reactions in the system (Figure S1).

**Figure 2:**
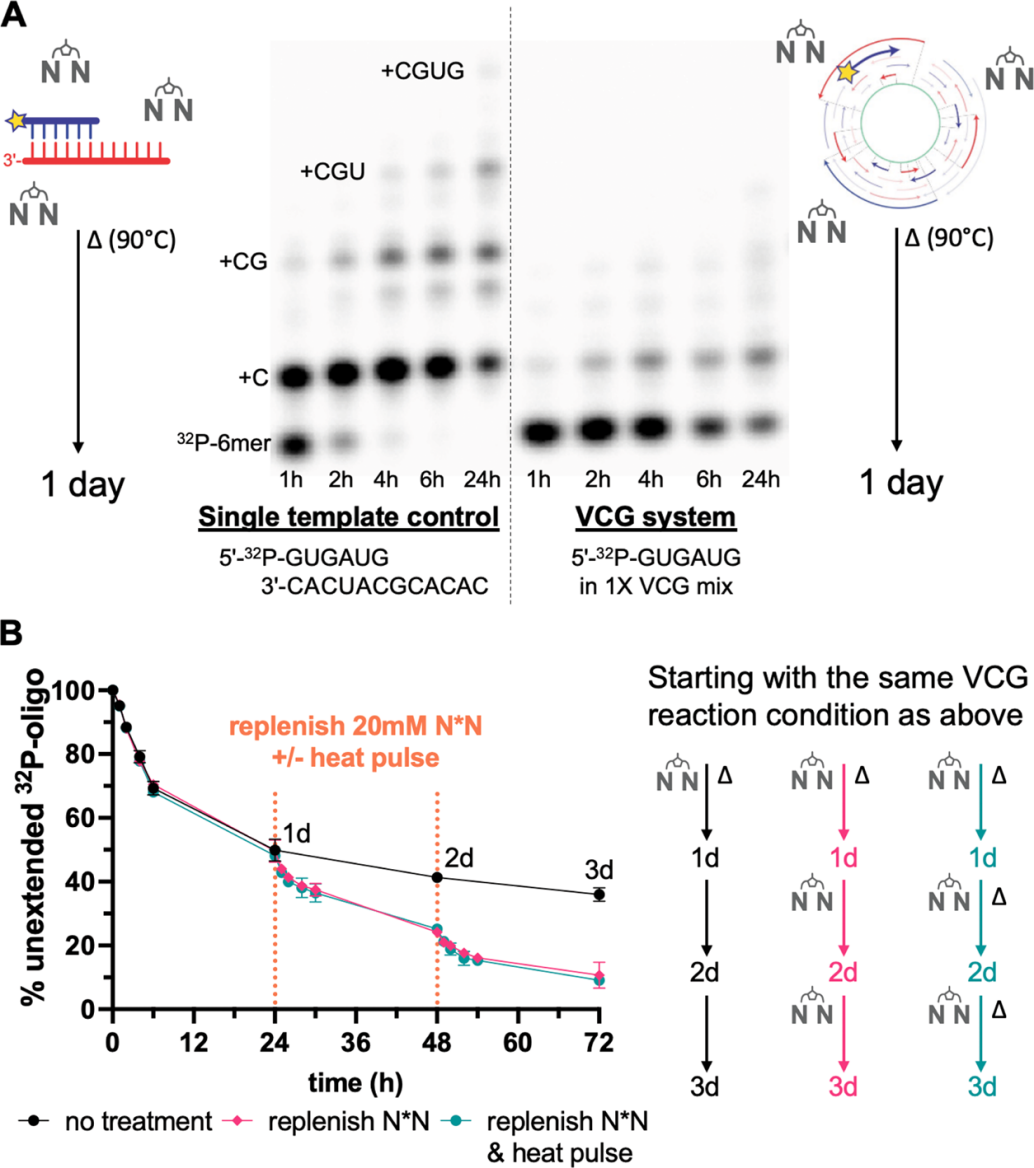
Demonstration of extension inside the virtual circular genome system. (A) Comparison between the VCG system and a single-template system, with schematic representations of the two experiments shown flanking the PAGE gel image. The VCG system contains 1 μM of all of the VCG oligos listed in Table S1. The single-template system contains 1 μM of the template and 1 μM of the primer. Extensions were monitored using trace 5′-^32^P-GUGAUG added to the reactions. The small VCG diagram is adapted from Figure 3 of ref (23) with permission under a Creative Commons Attribution 4.0 International License. Copyright 2021 Zhou et al.; Cold Spring Harbor Laboratory Press for the RNA Society. (B) Continuous VCG extension for 3 days, with and without periodic replenishment of 20 mM activated N*N and 90 °C heat pulses. The scheme on the right demonstrates different treatments for the three reactions. All reactions were conducted at room temperature, with 50 mM MgCl_2_, 200 mM Tris–HCl (pH 8.0), and 20 mM pre-equilibrated N*N.

We then set out to determine whether it is possible for oligonucleotides to be elongated by primer extension in the highly complex virtual circular genome system. We monitored the extension of individual labeled primers occurring within the mixture of 247 different VCG oligonucleotides (Figure 2A). We started by monitoring a single radiolabeled 6-mer oligonucleotide added in trace concentration (<0.05 μM) to a mixture of 1 μM of each VCG oligonucleotide, which we refer to as the 1× VCG mixture. About half of the initial radiolabeled 6-mer was extended to the corresponding 7-mer in 1 day. This rate of primer extension was much slower than in the positive control, in which the same labeled primer was incubated with only one complementary 12-mer template (1 μM). Nevertheless, this observation shows that a significant fraction of the VCG oligonucleotides anneal to form configurations that are productive for primer extension and that a fraction of these configurations exist for a time scale of hours to days.

We then asked what limits primer extension in the virtual circular genome system compared to the single-template system. One possibility is that fast equilibration of the oligonucleotides depletes available templates as they become sequestered in stable duplexes. Since only one heat pulse was applied to dissociate duplexes and initialize the process, if all oligonucleotides with melting points above room temperature quickly equilibrated back to form stable duplexes with their own complementary strands, then the labeled 6-mer would be rapidly dissociated from any suitable templates for primer extension. An alternative extreme possibility would be the continued but very slow rearrangement of the initially formed oligonucleotide complexes. If all oligonucleotide configurations after the initial heat pulse were locked in place, then any radiolabeled 6-mer trapped in an unproductive configuration would not be able to shuffle into a productive configuration, and primer extension would cease after all initially productive configurations had become extended. However, it is unlikely for a 6-mer with an estimated *k*_off_ of ∼19 s^–1^ to its complementary strand^29^ to bind so tightly that it could not either spontaneously dissociate from its template or be strand displaced by another longer complementary oligonucleotide. The resulting free 6-mer could then anneal to a new template, where it would have another opportunity to be extended.

Besides the equilibration and rearrangement rates, another potential limiting factor in the VCG system is simply the proportion of productive configurations at any given time. Since nonenzymatic templated extension requires at least two open nucleotide binding sites downstream of a template-bound primer for efficient reaction, any other kinetically trapped configurations will block templated extension (Figure 1B). Unlike the single-template system, where most primers can form the appropriate primer–template complex and therefore be extended, many of the oligonucleotides in the VCG system will be at least initially bound in unproductive configurations. Because the initial rate of primer extension in the VCG mix is slower than the rate in the single-template control, we hypothesize that the initial limiting factor for fast primer extension is the proportion of productive configurations and that slow equilibration in the complex virtual circular genome system as well as the ongoing hydrolysis of the activated species are responsible for the subsequent continuing decline in the rate of primer extension.

### Rearrangement and Equilibration of the Base-Paired Configurations in the VCG System

To test the idea that continued spontaneous shuffling of productive configurations might be occurring, we allowed the same primer extension reaction to continue for an extended time without any external treatments. Remarkably, template-directed primer extension continued for at least 3 days at an ever-declining rate (Figure 2B). This result suggests that at least a fraction of the oligonucleotide complexes were still shuffling and acting as templates for primer extension after 3 days. However, we suspected that the declining primer extension rate was also partially due to a declining concentration of activated species (N*N bridged dinucleotides) available at later times because of their relatively rapid hydrolysis under primer extension conditions (∼85% hydrolysis in 1 day) (Figure S2). Therefore, we performed a similar 3-day VCG reaction with replenishment of N*Ns each day. These freshly supplied activated species boosted the extent of primer extension in the VCG system, suggesting that a significant proportion of productive oligonucleotide configurations were still present in the system after 3 days. Having established that replenishment of activated nucleotides allows for continued primer extension, we then asked whether additional thermal cycling at later time points could improve primer extension by shuffling the base-paired configurations of the VCG oligonucleotides. To our surprise, when additional heat pulses were performed just prior to each N*N replenishment, no significant improvement in primer extension was observed. We speculate that the medium-sized oligonucleotides in the VCG system were probably shuffling well enough at room temperature to continuously generate productive configurations that additional heat pulses to reset the system did not induce significant improvement.

**Figure 3:**
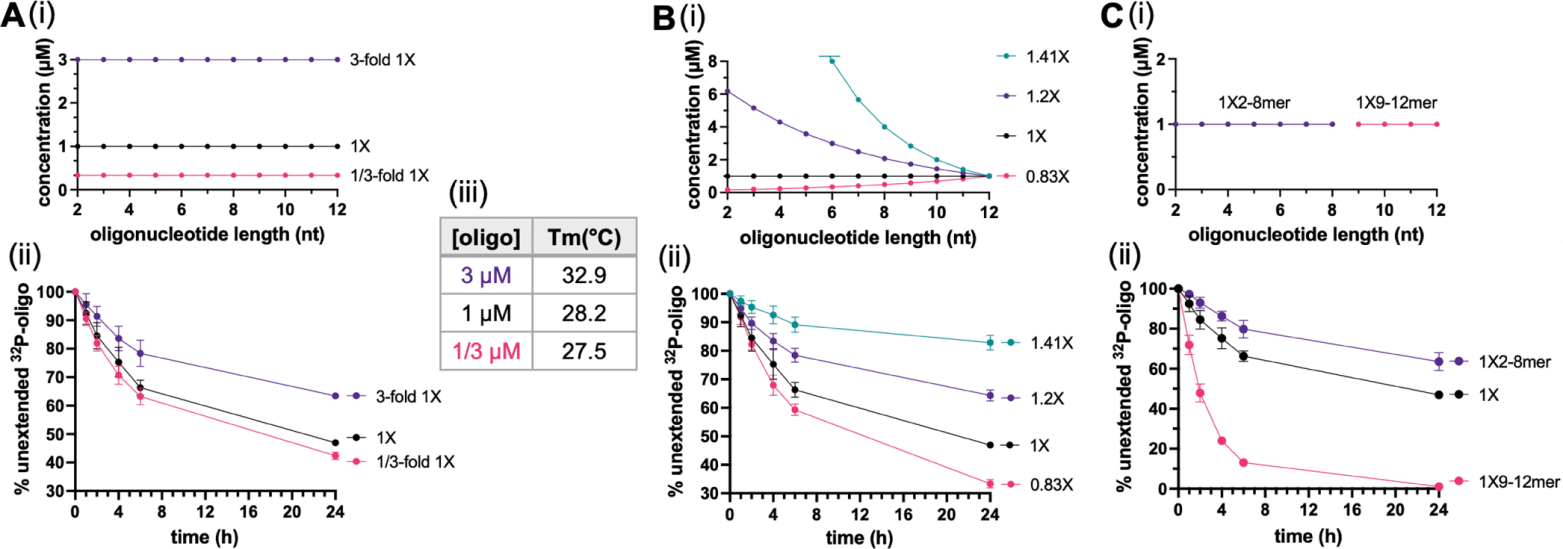
Virtual circular genome extension with different oligonucleotide compositions. (A) VCG extension when concentrated vs diluted. (i) Concentration of each oligonucleotide at the indicated length. (ii) VCG extension measured by % unextended 5′-^32^P-GUGAUG. (iii) Melting temperature of p-GUGAUG measured as a function of concentration in the primer extension buffer. (B) VCG extension at different concentration gradients. The concentration gradient is expressed as [(pN)_i_]/[(pN)_i+1_], starting at 1 μM of each 12-mer. (i) Oligonucleotide concentrations in each gradient. The concentrations of 2–5-mer in 1.41× exceed the *y*-axis limit. See Table S2 for all concentrations. (ii) VCG extension under different concentration gradients. (C) Extension in partial VCG mixtures containing only the longer or shorter oligomers. (i) Oligonucleotide composition in the partial VCG system. (ii) VCG extension in the partial system. All reactions were measured by the extension of 5′-^32^P-GUGAUG (<0.05 μM) conducted at room temperature, with 50 mM MgCl_2_, 200 mM Tris–HCl (pH 8.0), and 20 mM pre-equilibrated N*N. See Table S2 for detailed oligomer concentrations in different VCG mixtures.

Given the remarkably prolonged equilibration process in the VCG model, we asked if system-wide changes in oligonucleotide concentrations would impact the observed extent and rate of primer extension. Diluting or concentrating the entire VCG oligo mixture will affect the concentration of every oligonucleotide complex in the system by affecting the association rate for duplex formation. Although one might expect that dilution and hence weaker binding of the short 6-mer primer to templates would result in reduced primer extension, what we observed was the opposite. Under the same reaction conditions, a less concentrated VCG mixture exhibited faster primer extension and a greater yield of the extended product (Figure 3A). We suggest that the lowered concentration of short oligonucleotides allowed for a greater initial fraction of productive configurations and that the slower association rate for duplex formation allowed newly opened templates to remain available for the primer extension for a longer time. This result suggests that concentration fluctuations could facilitate the continued rearrangement of oligonucleotide configurations in the VCG mix. Changes in oligonucleotide concentration can also be interpreted in terms of concentration-dependent changes in duplex *T*_m_. A more dilute VCG mix implies a lower effective *T*_m_ for all oligonucleotide duplexes, which could facilitate continued shuffling of base-paired configurations. As a point of reference, we measured the melting temperature of our 6-mer primer and its complement at three different concentrations in a primer extension buffer to demonstrate this relationship (Figure 3A(iii)). A three-fold decrease in concentration led to a 1 °C decrease in *T*_m_, and even this modest effect was enough to lead to a noticeable increase in primer extension.

In a further attempt to manipulate the proportion of productive configurations in the VCG system, we adjusted the concentrations of the VCG oligonucleotides in a length-dependent manner. We reasoned that if, on average, elongation by primer extension is slow, as we observed, then a length-dependent concentration gradient might emerge, with shorter oligonucleotides being more abundant than longer oligonucleotides. For the following experiments, we made the simplifying assumption of an exponential gradient of length distribution, where the concentration gradient is defined as [(pN)_i_]/[(pN)_i+1_]. For example, a 1.41× VCG system with 1 μM of each 12-mer contains 1.41 μM of each 11-mer and 2 μM of each 10-mer. Table S2 lists the concentration of each oligonucleotide as a function of length in the different concentration gradients that we used for the following experiments. As previously noted, with a 2× concentration gradient, the primer extension of all oligonucleotides by one nucleotide on average results in duplication of the entire population, i.e., one round of replication. Similarly, a 1.41× (≈√2) gradient requires an average of 2 nucleotides and a 1.2× (≈4√2) gradient requires approximately 4-nt of primer extension for one round of replication.^23^

Experimentally, we observed that a steeper concentration vs the length gradient leads to a significantly slower rate of extension of a labeled 6-mer primer (Figure 3B). We interpret this effect as being due to increased competition for binding to the limited concentration of longer oligonucleotides, which are expected to be better templates as they are long enough to provide binding sites for a primer, a bridged dinucleotide substrate, and a downstream helper. The ratio of a 6-mer primer to a 12-mer template in a 1× concentration gradient is 1:1, but this ratio increases to 8:1 in the 1.41× and 64:1 in a 2× concentration gradient. As a result, the fraction of the 6-mer primer that is able to bind to a longer template oligonucleotide is lower with a steeper gradient. Thus primer extension, expressed as a fraction of the input primer, is decreased; however, it should be noted that the total amount of the extended primer is increased. For example, while the 1× gradient can produce 1 μM × 53% = 0.53 μM of newly extended 7-mer in one day, the 1.41× gradient can produce up to 8 μM × 18% = 1.44 μM, almost tripling the amount. The effect of the concentration gradient on the extension rate is seen with oligonucleotides of different lengths. We measured the extension of 8-, 10-, and 12-nt primers, and in all cases, the fraction of primer extended vs time was higher in a VCG mix with a shallower concentration vs length gradient (Figure S3). We also tested a 0.83× gradient, where longer oligonucleotides are present at higher concentrations than shorter oligonucleotides. With this reverse gradient, we observed a faster rate of primer extension than in a 1× gradient, presumably due to the higher availability of longer oligonucleotides as good templates.

To further investigate the factors controlling the rearrangement of base-paired configurations, we explored partial VCG systems where only the shorter or longer VCG oligonucleotides were supplied. An optimal template for primer extension requires sufficient complementarity to the primer for stable binding and at least two additional unpaired nucleotides downstream of the primer to act as the binding site for an activated bridged dinucleotide. For our 6-mer primer, an optimal template would need to be at least 8-nt long. We first examined a partial VCG system consisting of only 2- to 8-mer oligonucleotides. In this system, only one of the 24 8-mers would be an optimal template for the radiolabeled 6-mer. We observed a slower initial rate of primer extension and a lower extent of primer extension at 24 h in the 2–8-mer partial VCG system than in the complete system (∼36 vs 53%), presumably because of the low proportion of the productively arranged 6-mer primer at any given time point (Figure 3C). However, even though the rate was low, this observation suggests that even a VCG system with an 8-nt genome allows extensions. On the other hand, the 9–12-mer partial VCG system, which contains only the longer subset of oligonucleotides, shows extremely good primer extension, with essentially complete primer extension by one or more nucleotides in one day. Because all of these longer oligonucleotides are present together with their complementary strands in the VCG system, we initially expected that the rapid formation of stable duplexes would prevent significant primer extension. Since not all of the radiolabeled 6-mer could be in a productive configuration after the initial heat pulse, the fact that primer extension continued until all of the 6-mer primer had been extended implies that rearrangements of base-paired configurations were happening in the VCG system for these 9–12-nt oligonucleotides at room temperature.

### Extension of Oligonucleotides of Different Lengths in the VCG System

**Figure 4:**
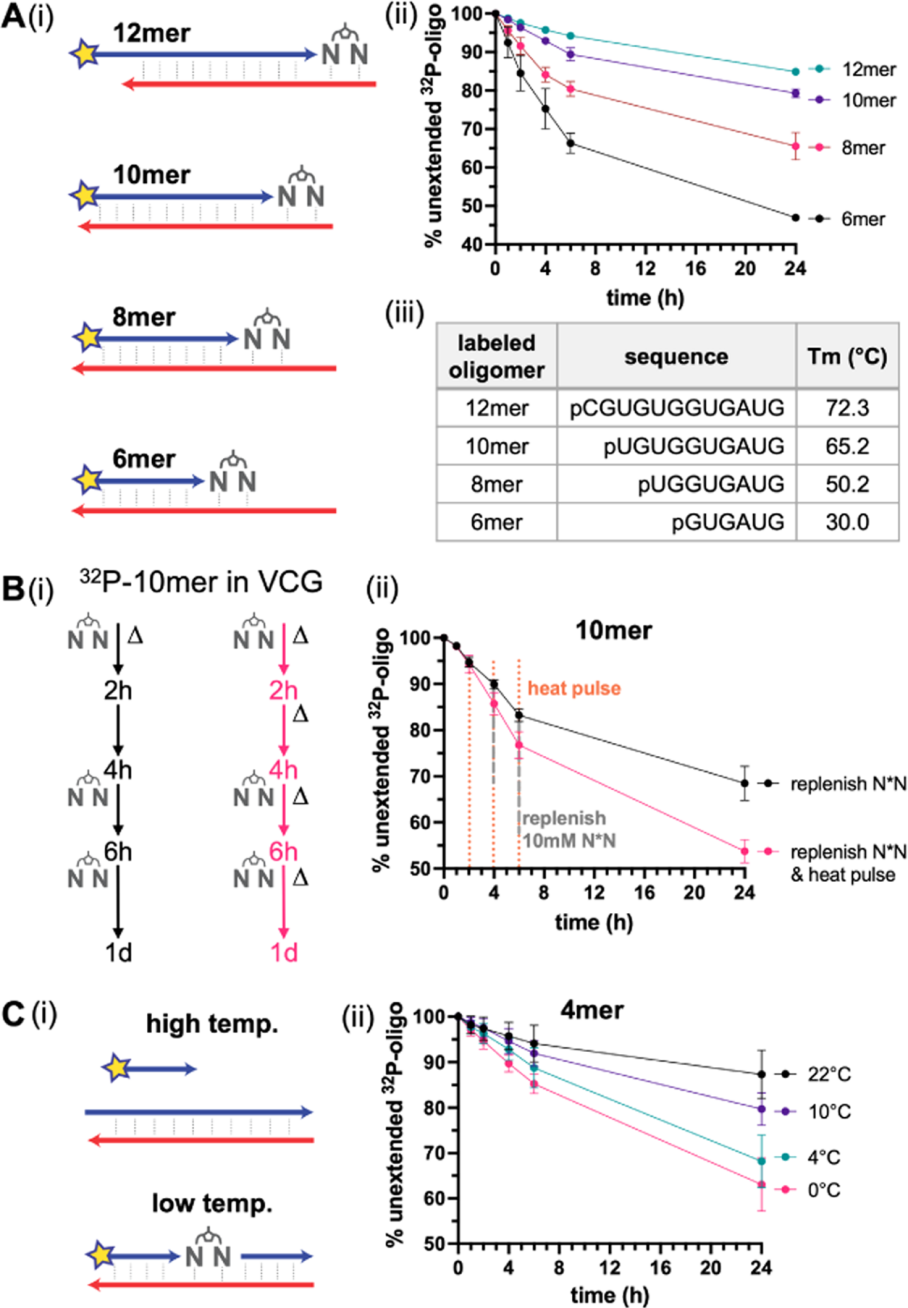
Length dependence and temperature effect on the primer extension in the 1× VCG system. (A(i)) Schematic representation of possible base-paired configurations between radiolabeled oligonucleotides of varying lengths and complementary 12-mers. (ii) Extension of oligonucleotides with different lengths in the 1× VCG oligo mix, represented by the percentage of unextended 5′-^32^P-labeled oligonucleotide over time. (iii) Sequences of the labeled oligomers and their melting temperatures, measured in the primer extension buffer. (B) Heat pulses facilitate the continuous VCG extension of a 10-mer (5′-^32^P-UGUGGUGAUG). (i) Experimental scheme. (ii) Measured extension with or without the heat pulses. The heat pulses were performed by 10 s of 90 °C heating, followed by immediate 1 min cooling on ice. The replenishments were performed by adding 10 mM of equilibrated and lyophilized N*N powder. (C) Lower temperature facilitates the VCG extension of a 4-mer (5′-^32^P-GAUG). (i) A scheme showing that a tetramer in the VCG has a higher chance to anneal to a complementary strand at lower temperatures. (ii) 4-mer extension in VCG at different temperatures. All reactions were conducted in 1× VCG with 200 mM Tris–HCl (pH 8.0), 50 mM MgCl_2_, and an initial addition of 20 mM N*N.

The length of an oligonucleotide in the VCG system is likely to affect both its initial likelihood of annealing in a productive configuration as well as the dynamics of the exchange processes that would allow for continued primer extension. We, therefore, determined primer extension rates for a series of oligonucleotides of different lengths (Figure 4A). To avoid the effects of differing sequences at the 3′-end of the primer, we used a set of oligonucleotides with the same 3′-end as the 6-mer primer used above and varied only the 5′-end. Initially, we expected that longer oligonucleotides might show faster initial rates of primer extension since they would be able to bind more strongly to longer templates. We also expected slower long-term rates of primer extension since they would be more likely to become sequestered in stable, unproductive configurations that would be unable to exchange into new productive configurations. Surprisingly, we observed a progressive decrease in both the initial and long-term rates of primer extension as oligonucleotide length increased from 6 to 8, 10, and then to 12 nucleotides. We suggest that both of these effects stem from a decreased probability of forming productive configurations. The melting temperatures of these oligonucleotides, when paired with their perfect complements, increase significantly with length (Figure 4A(iii)). This greater duplex stability is likely to decrease the spontaneous shuffling of paired configurations in the VCG system, decreasing the rate of primer extension at long times. Why longer primers are extended more poorly initially is less clear but could potentially be due to occupancy by pairs of shorter oligonucleotides, preventing the formation of productive configurations. Alternatively, toehold-mediated branch migration may lead to rapid loss of productive configurations, thereby decreasing primer extension even at early times.

To facilitate the shuffling of the longer oligonucleotides for continuous elongation, we tested the effect of periodic temperature fluctuations on the extension of the 10-mer primer. After an initial high-temperature pulse to initialize the system, three additional high-temperature pulses (90 °C for 10 s) were applied every 2 h to shuffle the oligonucleotide configurations. Fresh activated N*Ns were added after the second and third high-temperature pulses to counter the effects of hydrolysis since about half of the initial bridged dinucleotides had already hydrolyzed after 4 h (Figures S1 and S2). A clear improvement in the extent of 10-mer extension was observed with the extra high-temperature pulses, demonstrating the importance of temperature fluctuations for the continued elongation of longer oligonucleotides in the VCG system (Figure 4B). As expected, the improvement in primer extension was even greater when fresh N*N substrates were added after each high-temperature pulse.

In contrast to the requirement of high-temperature fluctuations for primer extension of longer oligonucleotides, we have found that shorter oligonucleotides can only be extended at lower temperatures. When a 4-mer primer is radiolabeled and monitored in the VCG mix at room temperature (22 °C), we observe only minimal primer extension. In addition to the low yield, many of the extended products formed were incorrect (Figure S4B). We first hypothesized that in the VCG mix, much of the 4-mer was not bound to any template most of the time at room temperature and that the observed extension arose primarily through untemplated extension. However, a control experiment showed that the 4-mer can extend efficiently on a single template at room temperature (80% at 24 h), although the extent of primer extension does improve markedly at lower temperatures (Figure S4A). Therefore, the poor extension of the 4-mer primer in the VCG system is not solely due to poor binding. We speculated that rapid dissociation of the 4-mer from a template strand, followed by template occupancy by a competing oligonucleotide, would prevent primer extension (Figure 4C(i)). We, therefore, tested the effect of reducing the temperature on 4-mer extension yield in the VCG system. As temperatures decreased, we observed increased correct extension and decreased misincorporation (Figures 4C(ii) and S4B). The remarkably improved yield and fidelity suggest that primer extension of the shorter oligonucleotides in the VCG system requires a lower temperature to prevent rapid loss of productive configurations.

### Fidelity in the Virtual Circular Genome Scenario

The significant degree of misincorporation observed with the 4-mer at room temperature drew our attention to the possibility of untemplated extension in the VCG system. The untemplated extension could result not only from unbound oligonucleotides but also from some of the unproductive configurations in the VCG system. As shown in Figure 1B, many unproductive configurations have either an overhanging or blunt 3′-end that can potentially be subject to untemplated extension. Moreover, the 5′-phosphate of both free oligonucleotides and some template-bound oligonucleotides may also react to form 5′-5′-pyrophosphates. Indeed, polyacrylamide gel electrophoresis (PAGE) analysis of the extension of the 6-mer primer in the VCG system (Figure 2A) clearly shows that several products are formed that are not seen in the single-template system, suggesting that these misincorporations most likely derive from processes other than templated primer extension. Interestingly, increasing the concentration of the bridged dinucleotides enhanced the synthesis of these incorrect products but did not significantly improve the correct templated primer extension reaction (Figure S5).

To identify the sources of these misincorporations, we first examined the untemplated extension of specific oligonucleotides in the presence of all possible activated bridged dinucleotides. In the VCG system, because every oligonucleotide exists in the presence of its partially and fully complementary strands, blunt ends can form at either end of the oligonucleotide. Therefore, we tested both single-stranded RNAs of different lengths and the corresponding double-stranded duplexes for untemplated extension. To our surprise, we observed enhanced untemplated extension with blunt-ended species (Figure S6).

Because of the limited ability of PAGE analysis to resolve different products of untemplated extension, we determined the extent and regioselectivity of untemplated extension by supplying only one bridged homo-dinucleotide at a time (Figure S7). The identity of each extended product was determined by comparison with authentic radiolabeled samples (see Materials and Methods for the synthesis of standards). Untemplated oligonucleotide polymerization has long been known to favor 2′- over 3′-extension due to the greater nucleophilicity of the 2′-hydroxyl group, and the formation of 5′-5′ pyrophosphate products is known to be an unavoidable byproduct of reactions with nucleotide phosphorimidazolides.^30,31^ In our examination of untemplated extension, we also observed a predominance of products with nucleotides added at either the 2′-OH or 5′-phosphate. Blunt-ended duplex oligonucleotides appear to be particularly prone to nucleotide addition to the 2′-hydroxyl, especially with G (Figure S7). In addition to the untemplated extension of single-stranded and blunt-end RNAs, we also examined the primer extension of the labeled 6-mer primer in the VCG mix in the presence of only one imidazolium-bridged homo-dinucleotide at a time. Note that correct templated extension, in this case, requires a C*G bridged dinucleotide. In the absence of this fully complementary substrate, the products of the primer extension were quite similar to those of the untemplated reactions, with most of the elongations being at the 2′- or 5′-end. When supplied with a C*C bridged dinucleotide, the observed correct 3′-extension with C probably resulted from the substrate binding to the template with a downstream C:C mismatch. Interestingly, we observed less extension with bridged homo-dinucleotides in the VCG system than with an isolated duplex, especially when G*G was supplied. This finding suggests that annealing of the oligonucleotides in the VCG mix results in a low proportion of blunt-ended duplexes, as might be expected since there are many more annealed configurations with 5′- or 3′- overhangs than blunt-ended configurations.

**Figure 5:**
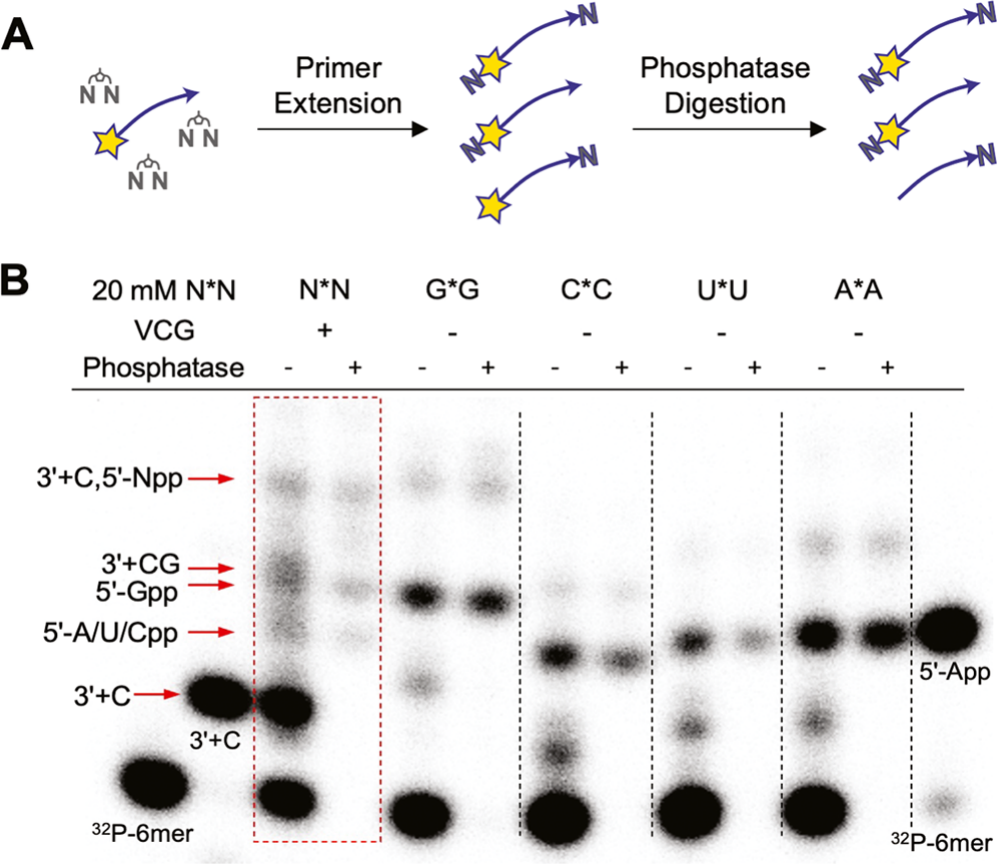
Detection of 5′-pyrophosphate-capped oligonucleotides. (A) Schematic representation of the phosphatase deradiolabeling of the extension products. The 5′-^32^P labels were shown as stars. The 5′-^32^P-oligonucleotides would be dephosphorylated while the 5′-Np^32^P-oligonucleotides would be protected. (B) PAGE gel analysis of the extension products with or without phosphatase digestion. The VCG extension was performed with the 1× VCG mixture and 20 mM N*N, while the untemplated reactions were performed with 1 μM 6-mer and 20 mM of the indicated imidazolium-bridged homodimers. See Figure S7B for more details. All reactions were run at room temperature for 24 h. Phosphatase-digested products were loaded at the same concentration as the untreated sample. Authentic samples were run alongside the PAGE gel and are indicated in the figure.

To better identify misincorporation events, we aligned the gel-separated VCG extension products with the individual untemplated reaction products; we also used phosphatase digestion to distinguish between the 5′- (which protects the ^32^P-labeled 5′-phosphate from digestion) and 2′/3′-extension (Figures 5 and S7). This assay showed that most of the apparent misincorporations in the VCG reaction were, in fact, due to 5′-nucleotide-pyrophosphate formation. We could not quantify how much of each pyrophosphate is formed because primers with 5′-App-, 5′-Upp, and 5′-Cpp- have almost identical gel mobilities. However, in the reactions with single N*N substrates, A*A led to more formation of 5′-App-oligo products than the corresponding products with C*C, U*U, and G*G. It is also possible that the 5′-Gpp extension of the specific radiolabeled primer we used could be template-directed in the VCG mix. The 2′ + A and 2′ + U products have similar gel mobility to the correct (templated) 3′ + C product. However, we believe that there is little 2′-extension with A and U because no significant amount of 2′ + C or 2′ + G products was formed (Figure S7B). The small amount of slowly migrating products in the VCG primer extension reaction that survived the phosphatase digestion likely corresponds to the 5′-Npp extension of the correct 3′ + C product. As a result, most of the misincorporations we observed in the VCG systems appear to result from the 5′-5′-pyrophosphate formation. We note that 5′-Npp-capped oligonucleotides can still act as fully functional primers and templates in the VCG system; the accumulation of 5′-Npp-oligonucleotides could also provide a selective advantage for the evolution of ribozyme ligases that use such molecules as substrates.^32^

### Potential Strategies to Enhance Extensions in a VCG System

Having characterized the basic kinetics and fidelity of primer extension in our model VCG system, we asked what factors might further increase the rate and yield of primer extension. Considering the rapid hydrolysis of imidazolium-bridged substrates, an efficient method for in situ activation would likely be extremely beneficial. This ideal approach would lead to efficient activation of both monomers and oligonucleotides, as this would allow for the formation of monomers bridged to oligonucleotides, which we have previously shown to be optimal substrates for primer extension.^19^ We, therefore, asked whether preactivation of the VCG oligonucleotide mix would enhance primer extension by allowing for the formation of monomer-bridged-oligonucleotide intermediates.

We began by testing whether an activated trimer helper could accelerate the extension of our labeled 6-mer primer in the VCG system. We prepared the activated trimer *GUG and doped it at increasing concentrations into the partial 9–12-mer VCG system. Following the addition of activated monomers or bridged dinucleotides, this trimer can form the highly reactive C*GUG intermediate in situ. The higher affinity and greater preorganization of this substrate facilitate the +C extension of the ^32^P-labeled 6-mer primer. Previous kinetic measurements have shown that a similar monomer-bridged-trimer (specifically, A*CGC) has a *K*_M_ of 40 μM and a Vmax approaching 1 min^–1^ on a single template.^19^ When we added the *GUG helper together with an equilibrated mix of imidazolium-bridged dinucleotides to the partial 9–12-mer VCG oligonucleotides, we observed significant acceleration of primer extension when it was present at a concentration (∼50 μM) closer to the estimated *K*_d_ of C*GUG. Moreover, primer extension in the partial VCG system supplied with 100 μM *GUG can be almost as fast as the one-template positive control (Figure S8).

**Figure 6:**
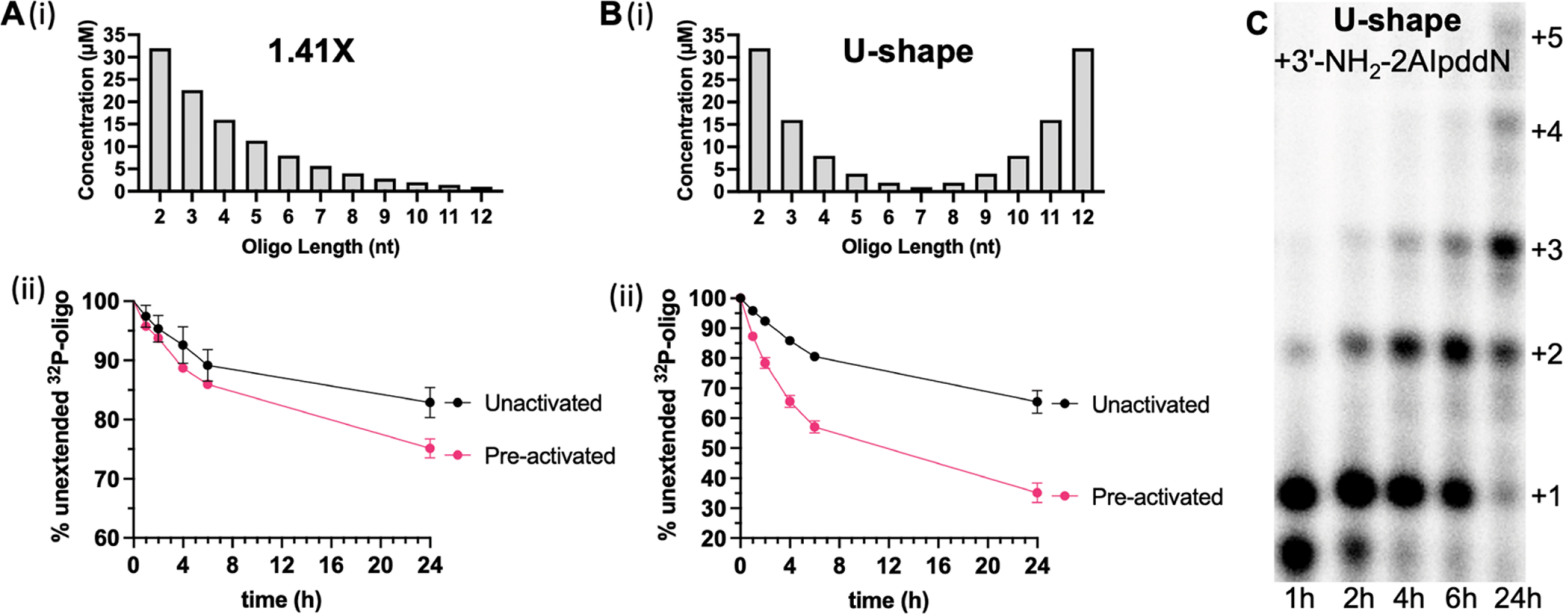
Demonstration of possible strategies to improve VCG extension (A–B) Significantly enhanced VCG extension after preactivation with either a 1.41× or U-shaped gradient. (i) Oligo concentration of each length. (ii) Comparison between the extensions of 5′-^32^P-GUGAUG inside the VCG system with or without preactivation. (C) Faster extension in a U-shaped VCG mix with the more reactive 3′-NH_2_-2AIpddN modification as a model system.

Encouraged by the observed benefit of adding a single activated helper oligonucleotide, we asked whether activating the entire set of VCG oligonucleotides would also help monomer-bridged-oligonucleotides form in situ and thus enhance primer extension. An important concern is that the excess amount of 2-aminoimidazole required for efficient activation will also reduce the formation of imidazolium-bridged substrates. To avoid this problem, we used stochiometric 2AI to activate a concentrated set of VCG oligonucleotides in a partially frozen reaction mixture at −15 °C and then thawed and diluted the mixture to allow primer extension to occur at room temperature. The partial freezing process served to concentrate the solutes in the liquid eutectic phase between the pure ice crystals. We have previously used this approach to enable efficient in situ activation of imidazolium-bridged species for nonenzymatic template copying.^33^ Here, we used the non-prebiotic 1-ethyl-3-(3-dimethylaminopropyl)carbodiimide (EDC) as the coupling reagent for activation for ease of handling, but similar activation chemistry can be performed using the more prebiotically plausible methyl isocyanide. A control NMR experiment with a single dinucleotide demonstrated almost complete activation under the same conditions (Figure S9). After overnight eutectic phase activation, the reaction was warmed to room temperature, diluted into the primer extension buffer, and a ^32^P-labeled 6-mer primer was added. We started by activating the 1.41× VCG mix, in which short oligonucleotides are present at higher concentrations than the longer oligonucleotides. We observed significant enhancement of primer extension (Figure 6A) even though the concentrations of the short oligonucleotides were still far below the *K*_d_ of the corresponding monomer-bridged-oligonucleotides.^19^ When we activated the 1× VCG system, we observed no rate enhancement, probably because the concentration of the short helper oligonucleotides was too low.

We then reasoned that the optimal concentration vs length distribution might be more complex than a simple exponential gradient. Clearly, short oligonucleotides must be present close to their *K*_d_ to have a significant effect on primer extension. On the other hand, medium-length oligonucleotides are elongated most rapidly and therefore might be present at lower steady-state concentrations, while longer oligonucleotides might accumulate and reach higher concentrations. We therefore prepared and activated a VCG mix with a U-shaped concentration vs length distribution (Table S2). We were pleased to observe improved primer extension in this system, with about 70% of the labeled primer being extended by one or more nucleotides in less than one day (Figure 6B).

Finally, we asked how primer extension in the VCG system would be affected if the reaction kinetics were improved. To do this, we employed a ^32^P-labeled 6-mer primer terminated with a highly reactive 3′-amino-2′,3′-dideoxy-ribonucleotide, and similarly modified 2-aminoimidazole activated mononucleotides (3′-NH_2_-2AIpddNs). Although such nucleotides may not be prebiotically plausible, they provide an excellent model system for the simulation of nonenzymatic RNA copying under conditions leading to enhanced rates of primer extension, such as might be achieved, e.g., by a prebiotic catalyst or improved conditions for chemical RNA copying. By employing the highly nucleophilic 3′-amino group, we were able to observe ∼60% +1 primer extension in just 1 h, with almost complete +1 or greater extension by 4 h and a low fraction of misincorporations (Figure 6C). Remarkably, an average extension of ∼ +3 nucleotides was observed by 24 h, consistent with the spontaneous shuffling of partially base-paired configurations continuing for many hours.

## Discussion

We first proposed the virtual circular genome model^23^ as a theoretical means of overcoming the barriers to prebiotically plausible RNA replication. Replication in the VCG model does not require the specific primers needed for replication of a linear genome, and the distributed nature of the copying processes is expected to impart resilience to chemical processes that modify or block the 5′- or 3′- ends of individual oligonucleotides. Importantly, the repeated shuffling of base-paired configurations of annealed oligonucleotides was proposed as a means of overcoming the block to replication imposed by rapid strand annealing. However, experimental tests of this model were clearly needed, as template copying by primer extension has previously been examined only in highly simplified model systems. Our studies show that primers of different lengths can indeed be extended by template copying with a significant rate, extent, and fidelity in a model VCG system, suggesting that under appropriate environmental conditions, replication in the VCG mode may be possible.

Perhaps the most surprising aspect of our results is the prolonged time scale (>1 day) over which primer extension in the VCG mixture continues. We interpret the extended time scale of primer extension as reflecting the very slow equilibration of the VCG oligonucleotides. The shuffling of a simpler set of DNA oligonucleotides for template copying and replication has been studied before,^34,35^ and our study provides further insights into how a complex mixture of RNAs would slowly equilibrate to enable nonenzymatic replication. The very large number of competing base-paired configurations of VCG oligonucleotides may prevent rapid equilibration to fully base-paired duplexes, thus allowing for the continued shuffling of partially base-paired configurations. At any given time, only a fraction of these configurations is productive for primer extension, while others are not. If unproductive configurations can rearrange by dissociation, exchange, or strand displacement, new productive configurations may continue to arise, enabling the observed extended time course of primer extension.

We have found that oligonucleotides that were both longer (8, 10, and 12-nts) and shorter (4-nts) than the 6-mer primer exhibited slower and less extensive primer extension. Short pulses of high temperature partially rescued the poor extension of the longer primers, suggesting that these oligonucleotides tend to become trapped in stable unproductive configurations that can be disrupted and exchanged during exposure to elevated temperatures. In contrast, the shorter 4-nt oligonucleotide required a lower temperature for optimal primer extension, in part due to the weaker binding to template strands but also in part due to the greater lability of productive configurations involving a base-paired 4-nt primer. These divergent temperature requirements for the primer extension of oligonucleotides of different lengths imply that repeated cycles of RNA replication would only be possible in a fluctuating environment. For example, changing temperature, pH, or salt concentrations could trigger ongoing shuffling of the annealed configurations of the VCG oligonucleotides.

The observed variation in the rate of primer extension with primer length has implications for the steady-state length vs concentration profile. In our original model, we assumed for simplicity an exponential concentration vs length gradient, i.e., a constant [length *n*]/[length *n* + 1] ratio. However, if short and long oligonucleotides are elongated more slowly than medium-length oligonucleotides, both short and long oligonucleotides would tend to accumulate, while medium-length oligonucleotides would be rapidly extended, resulting in a more U-shaped concentration vs length distribution. The short oligonucleotides can be activated to form monomer-bridged-oligonucleotides that will facilitate faster extension, while the longer oligonucleotides are good templates for oligonucleotide extension. Further experiments will be required to determine the steady-state distribution of VCG oligonucleotides as a function of the length and sequence over multiple cycles of replication.

Given the observed advantage of activating the VCG oligonucleotides and the fast rate of hydrolysis of bridged N*N intermediates, some means of in situ activation will clearly be required to enable continued oligonucleotide elongation and thus complete cycles of replication. Our laboratory has recently demonstrated prebiotically plausible activation and bridge-forming chemistry that allows one-pot conversion of nucleotides to bridged dinucleotides with a high yield; however, this process requires repeated freeze-thaw cycles, which are known to disrupt vesicles.^33^ Therefore, a less disruptive process, compatible with vesicle integrity, may be required for VCG replication within protocells. Alternatively, if eutectic phase activation chemistry occurred in a distinct, separate environment, periodic melting could potentially release fresh activated nucleotides that could flow over a population of protocells and diffuse into the vesicles while hydrolyzed nucleotides diffuse out. In such a flow system, the free 2AI generated from the formation of imidazolium-bridged species could diffuse out of the vesicles, shifting the equilibrium inside the vesicles to favor the formation of 2AI-bridged dinucleotides and monomer-bridged-oligonucleotides.

Template copying in the VCG system must proceed with sufficient fidelity to allow the inheritance of useful amounts of information. For a ribozyme on the order of 50 nucleotides in length, this implies an error rate of roughly 2% or less. Examination of the PAGE gels used to monitor primer extension reactions in our model VCG system reveals the presence of bands that do not correspond in mobility to the correct products of primer extension. In principle, these bands could correspond to products of primer extension with an incorrect nucleotide, or to extension with a correct or incorrect nucleotide at the 2′-hydroxyl of the primer, or to the addition of a nucleotide at the 5′-end of the primer via a 5′-5′ pyrophosphate linkage, which could be formed by attack of the 5′-phosphate of the primer on the phosphate of an activated monomer. Our experiments clearly show that 5′-5′ pyrophosphate-capped oligonucleotides are generated during primer extension in the VCG system, especially from blunt-ended duplexes. One of the major benefits of the VCG system is that there is no defined start or end to the genomic sequence, and oligonucleotides with a 5′-cap can still act as primers or templates. Furthermore, the synthesis of 5′-5′ pyrophosphate-capped oligonucleotides suggests a straightforward way in which the evolution of ribozymes could potentiate replication. Pyrophosphate-capped oligonucleotides can be substrates for ligation by ribozyme ligases, much as modern DNA and RNA ligases utilize an adenosine-5′-5′-pyrophosphate-activated substrate.^36^ Our lab has previously evolved a ribozyme ligase that catalyzes the ligation of adenylated RNAs to demonstrate the prebiotic possibility of such a mechanism.^32^

In addition to mutations induced by 3′-misincorporations, mispriming can also be a source of mutations. A vesicle membrane that would encapsulate the VCG system and separate it from the external environment could therefore be extremely beneficial. The uptake of short oligonucleotides, such as dimers and trimers, from the external environment should not cause problems, as even a 50-nt VCG would be likely to contain all di- and trinucleotide sequences. On the other hand, the uptake of longer mismatched oligonucleotides (5–8-nt) could be mutagenic. This may provide a useful constraint in defining the desirable properties of protocell membranes. Compartmentalizing each individual VCG system inside a protocell is thus necessary to prevent contamination of the VCG with random oligonucleotides that would lead to extensive mispriming.

Finally, we note that genome replication via the VCG model provides the raw materials necessary for spontaneous ribozyme assembly from oligonucleotides with lengths of roughly 10–20 nts. Partially overlapping pairs of such oligonucleotides can anneal with each other, after which loop-closing ligation can lead to the formation of stem-loop structures.^16^ The iteration of such processes could then lead to the assembly of complex structured RNAs, including ribozymes. Furthermore, the short oligonucleotides of the VCG could be substrates for ribozyme-catalyzed ligation,^7,8^ facilitating a transition from nonenzymatic replication to ribozyme-catalyzed RNA replication.

## Conclusions

We initially proposed the virtual circular genome (VCG) model as an approach to the nonenzymatic replication of RNA. The distributed nature of template copying in the VCG model circumvents problems associated with the replication of long linear or circular genomes. Experimental tests of the rate, extent, and fidelity of template copying are clearly required to assess the viability of the VCG model. Our initial experiments show that template-directed primer extension can indeed occur within a complex synthetic VCG oligonucleotide mixture, supporting our conjecture that a fraction of annealed configurations of VCG oligonucleotides would be productive for substrate binding and reaction. The surprisingly long time course of primer extension suggests that these annealed configurations continue to rearrange spontaneously for extended times, approaching the thermodynamic minimum of full base-pairing very slowly. Our hypothesis that an exponential oligonucleotide concentration vs length profile would facilitate rapid replication is not supported; rather, we find that a U-shaped profile is optimal for template copying. We conclude that very short oligonucleotides must be present at high concentrations approaching their *K*_d_s for template binding to act as effective primers and helpers, while a high concentration of the longest oligonucleotides is beneficial because they are the best templates. In contrast, a high concentration of medium-length oligonucleotides is counter-productive because they primarily act to occlude needed templates. We find that continued primer extension is enhanced by replenishment of hydrolyzed substrates, strongly suggesting that in situ activation will be required before cycles of RNA replication can be demonstrated in a VCG system. Overall, our experiments suggest that RNA replication via the VCG model may be possible, given appropriate activation chemistry and environmental fluctuations. Additional experiments will be required to determine whether a replicating VCG system can be maintained by feeding with activated monomers or whether an input of activated oligonucleotides is also required. We are currently exploring approaches to the computational modeling of VCG replication and to the experimental demonstration of VCG replication within model protocells.

## Supporting Information Available

Abbreviations; general information; selection of the VCG sequence; synthesis of activated nucleotides; NMR equilibration and hydrolysis of activated nucleotides; radiolabeled oligonucleotides; monitoring primer extension in the VCG oligonucleotide mixture; melting temperature measurements; supplementary Figures S1 to S9; and supplementary Tables S1, S2 (PDF)

## Abbreviations

VCG: virtual circular genome
2-AI or *: 2-aminoimidazole or 2-aminoimidazolium
NMR: nuclear magnetic resonance
PAGE: polyacrylamide gel electrophoresis
EDC: 1-ethyl-3-(3-dimethylaminopropyl)carbodiimide

## References

[ref1] Szostak J. W. The Eightfold Path to Non-Enzymatic RNA Replication. J. Syst. Chem. 2012, 3, 2 10.1186/1759-2208-3-2.

[ref2] Mariani A.; Bonfio C.; Johnson C. M.; Sutherland J. D. pH-Driven RNA Strand Separation under Prebiotically Plausible Conditions. Biochemistry 2018, 57, 6382–6386. 10.1021/acs.biochem.8b01080. 30383375 PMC6340128

[ref3] He C.; Lozoya-Colinas A.; Gállego I.; Grover M. A.; Hud N. V. Solvent Viscosity Facilitates Replication and Ribozyme Catalysis from an RNA Duplex in a Model Prebiotic Process. Nucleic Acids Res. 2019, 47, 6569–6577. 10.1093/nar/gkz496. 31170298 PMC6649698

[ref4] Ianeselli A.; Mast C. B.; Braun D. Periodic Melting of Oligonucleotides by Oscillating Salt Concentrations Triggered by Microscale Water Cycles Inside Heated Rock Pores. Angew. Chem. 2019, 131, 13289–13294. 10.1002/ange.201907909. PMC7616952 31322800

[ref5] Martin C.; Frenkel-Pinter M.; Smith K. H.; Rivera-Santana V. F.; Sargon A. B.; Jacobson K. C.; Guzman-Martinez A.; Williams L. D.; Leman L. J.; Liotta C. L.; Grover M. A.; Hud N. V. Water-Based Dynamic Depsipeptide Chemistry: Building Block Recycling and Oligomer Distribution Control Using Hydration–Dehydration Cycles. JACS Au 2022, 2, 1395–1404. 10.1021/jacsau.2c00087. 35783166 PMC9241005

[ref6] Ianeselli A.; Atienza M.; Kudella P. W.; Gerland U.; Mast C. B.; Braun D. Water Cycles in a Hadean CO_2_ Atmosphere Drive the Evolution of Long DNA. Nat. Phys. 2022, 18, 579–585. 10.1038/s41567-022-01516-z.

[ref7] Kreysing M.; Keil L.; Lanzmich S.; Braun D. Heat Flux across an Open Pore Enables the Continuous Replication and Selection of Oligonucleotides towards Increasing Length. Nat. Chem. 2015, 7, 203–208. 10.1038/nchem.2155. 25698328

[ref8] Salditt A.; Keil L. M. R.; Horning D. P.; Mast C. B.; Joyce G. F.; Braun D. Thermal Habitat for RNA Amplification and Accumulation. Phys. Rev. Lett. 2020, 125, 048104 10.1103/PhysRevLett.125.048104. 32794805

[ref9] Engelhart A. E.; Powner M. W.; Szostak J. W. Functional RNAs Exhibit Tolerance for Non-Heritable 2′–5′ versus 3′–5′ Backbone Heterogeneity. Nat. Chem. 2013, 5, 390–394. 10.1038/nchem.1623. 23609089 PMC4088963

[ref10] Gavette J. V.; Stoop M.; Hud N. V.; Krishnamurthy R. RNA–DNA Chimeras in the Context of an RNA World Transition to an RNA/DNA World. Angew. Chem., Int. Ed. 2016, 55, 13204–13209. 10.1002/anie.201607919. 27650222

[ref11] Kim S. C.; O’Flaherty D. K.; Giurgiu C.; Zhou L.; Szostak J. W. The Emergence of RNA from the Heterogeneous Products of Prebiotic Nucleotide Synthesis. J. Am. Chem. Soc. 2021, 143, 3267–3279. 10.1021/jacs.0c12955. 33636080

[ref12] Prakash T. P.; Roberts C.; Switzer C. Activity of 2′,5′-Linked RNA in the Template-Directed Oligomerization of Mononucleotides. Angew. Chem., Int. Ed. Engl. 1997, 36, 1522–1523. 10.1002/anie.199715221.

[ref13] Kim S. C.; Zhou L.; Zhang W.; O’Flaherty D. K.; Rondo-Brovetto V.; Szostak J. W. A Model for the Emergence of RNA from a Prebiotically Plausible Mixture of Ribonucleotides, Arabinonucleotides, and 2′-Deoxynucleotides. J. Am. Chem. Soc. 2020, 142, 2317–2326. 10.1021/jacs.9b11239. 31913615 PMC7577264

[ref14] Zhou L.; Kim S. C.; Ho K. H.; O’Flaherty D. K.; Giurgiu C.; Wright T. H.; Szostak J. W. Non-Enzymatic Primer Extension with Strand Displacement. eLife 2019, 8, e51888 10.7554/eLife.51888. 31702557 PMC6872209

[ref15] Zhou L.; O’Flaherty D. K.; Szostak J. W. Assembly of a Ribozyme Ligase from Short Oligomers by Nonenzymatic Ligation. J. Am. Chem. Soc. 2020, 142, 15961–15965. 10.1021/jacs.0c06722. 32820909 PMC9594310

[ref16] Wu L.-F.; Liu Z.; Roberts S. J.; Su M.; Szostak J. W.; Sutherland J. D. Template-Free Assembly of Functional RNAs by Loop-Closing Ligation. J. Am. Chem. Soc. 2022, 144, 13920–13927. 10.1021/jacs.2c05601. 35880790 PMC9354263

[ref17] Wu T.; Orgel L. E. Nonenzymatic Template-Directed Synthesis on Hairpin Oligonucleotides. 3. Incorporation of Adenosine and Uridine Residues. J. Am. Chem. Soc. 1992, 114, 7963–7969. 10.1021/ja00047a001. 11538876

[ref18] Walton T.; Zhang W.; Li L.; Tam C. P.; Szostak J. W. The Mechanism of Nonenzymatic Template Copying with Imidazole-Activated Nucleotides. Angew. Chem., Int. Ed. 2019, 58, 10812–10819. 10.1002/anie.201902050. 30908802

[ref19] Ding D.; Zhou L.; Giurgiu C.; Szostak J. W. Kinetic Explanations for the Sequence Biases Observed in the Nonenzymatic Copying of RNA Templates. Nucleic Acids Res. 2022, 50, 35–45. 10.1093/nar/gkab1202. 34893864 PMC8754633

[ref20] Duzdevich D.; Carr C. E.; Ding D.; Zhang S. J.; Walton T. S.; Szostak J. W. Competition between Bridged Dinucleotides and Activated Mononucleotides Determines the Error Frequency of Nonenzymatic RNA Primer Extension. Nucleic Acids Res. 2021, 49, 3681–3691. 10.1093/nar/gkab173. 33744957 PMC8053118

[ref21] Tupper A. S.; Higgs P. G. Rolling-Circle and Strand-Displacement Mechanisms for Non-Enzymatic RNA Replication at the Time of the Origin of Life. J. Theor. Biol. 2021, 527, 110822 10.1016/j.jtbi.2021.110822. 34214567

[ref22] Kristoffersen E. L.; Burman M.; Noy A.; Holliger P. Rolling Circle RNA Synthesis Catalyzed by RNA. eLife 2022, 11, e75186 10.7554/eLife.75186. 35108196 PMC8937235

[ref23] Zhou L.; Ding D.; Szostak J. W. The Virtual Circular Genome Model for Primordial RNA Replication. RNA 2021, 27, 1–11. 10.1261/rna.077693.120. 33028653 PMC7749632

[ref24] Ferris J. P.; Ertem G. Oligomerization of Ribonucleotides on Montmorillonite: Reaction of the 5′-Phosphorimidazolide of Adenosine. Science 1992, 257, 1387–1389. 10.1126/science.1529338. 1529338

[ref25] Ferris J. P.; Hill A. R.; Liu R.; Orgel L. E. Synthesis of Long Prebiotic Oligomers on Mineral Surfaces. Nature 1996, 381, 59–61. 10.1038/381059a0. 8609988

[ref26] Monnard P. A.; Kanavarioti A.; Deamer D. W. Eutectic Phase Polymerization of Activated Ribonucleotide Mixtures Yields Quasi-Equimolar Incorporation of Purine and Pyrimidine Nucleobases. J. Am. Chem. Soc. 2003, 125, 13734–13740. 10.1021/ja036465h. 14599212

[ref27] Kanavarioti A.; Monnard P. A.; Deamer D. W. Eutectic Phases in Ice Facilitate Nonenzymatic Nucleic Acid Synthesis. Astrobiology 2001, 1, 271–281. 10.1089/15311070152757465. 12448990

[ref28] Weimann B. J.; Lohrmann R.; Orgel L. E.; Schneider-Bernloehr H.; Sulston J. E. Template-Directed Synthesis with Adenosine-5′-Phosphorimidazolide. Science 1968, 161, 387 10.1126/science.161.3839.387. 5661298

[ref29] Todisco M.; Szostak J. W. Hybridization Kinetics of Out-of-Equilibrium Mixtures of Short RNA Oligonucleotides. Nucleic Acids Res. 2022, 50, 9647–9662. 10.1093/nar/gkac784. 36099434 PMC9508827

[ref30] Lohrmann R.; Orgel L. E. Preferential Formation of (2′-5′)-Linked Internucleotide Bonds in Non-Enzymatic Reactions. Tetrahedron 1978, 34, 853–855. 10.1016/0040-4020(78)88129-0.

[ref31] Kanavarioti A.; Lee L. F.; Gangopadhyay S. Relative Reactivity of Ribosyl 2′-OH vs 3′-OH in Concentrated Aqueous Solutions of Phosphoimidazolide Activated Nucleotides. Origins Life Evol. Biospheres 1999, 29, 473–487. 10.1023/A:1006540607594. 10573689

[ref32] Hager A. J.; Szostak J. W. Isolation of Novel Ribozymes That Ligate AMP-Activated RNA Substrates. Chem. Biol. 1997, 4, 607–617. 10.1016/S1074-5521(97)90246-5. 9281527

[ref33] Zhang S. J.; Duzdevich D.; Ding D.; Szostak J. W. Freeze-Thaw Cycles Enable a Prebiotically Plausible and Continuous Pathway from Nucleotide Activation to Nonenzymatic RNA Copying. Proc. Natl. Acad. Sci. U.S.A. 2022, 119, e2116429119 10.1073/pnas.2116429119. 35446612 PMC9169909

[ref34] Edeleva E.; Salditt A.; Stamp J.; Schwintek P.; Boekhoven J.; Braun D. Continuous nonenzymatic cross-replication of DNA strands with *in situ* activated DNA oligonucleotides. Chem. Sci. 2019, 10, 5807–5814. 10.1039/C9SC00770A. 31293769 PMC6568275

[ref35] Kühnlein A.; Lanzmich S. A.; Braun D. tRNA sequences can assemble into a replicator. eLife 2021, 10, e63431 10.7554/eLife.63431. 33648631 PMC7924937

[ref36] Orgel L. E. RNA Catalysis and the Origins of Life. J. Theor. Biol. 1986, 123, 127–149. 10.1016/S0022-5193(86)80149-7. 2442564

